# Lipophilicity Assessment of Ruthenium(II)-Arene Complexes by the Means of Reversed-Phase Thin-Layer Chromatography and DFT Calculations

**DOI:** 10.1155/2014/862796

**Published:** 2014-01-22

**Authors:** Khalil Salem A. M. Shweshein, Filip Andrić, Aleksandra Radoičić, Matija Zlatar, Maja Gruden-Pavlović, Živoslav Tešić, Dušanka Milojković-Opsenica

**Affiliations:** ^1^Faculty of Chemistry, University of Belgrade, Studentski Trg 12–16, P.O. Box 51, 11158 Belgrade, Serbia; ^2^Institute for Chemistry, Technology and Metalurgy, Center of Chemistry, University of Belgrade, Njegoševa 12, 11000 Belgrade, Serbia

## Abstract

The lipophilicity of ten ruthenium(II)-arene complexes was assessed by reversed-phase thin-layer chromatography (RP-TLC) on octadecyl silica stationary phase. The binary solvent systems composed of water and acetonitrile were used as mobile phase in order to determine chromatographic descriptors for lipophilicity estimation. Octanol-water partition coefficient, log*K*_OW_, of tested complexes was experimentally determined using twenty-eight standard solutes which were analyzed under the same chromatographic conditions as target substances. In addition, *ab initio* density functional theory (DFT) computational approach was employed to calculate log*K*_OW_ values from the differences in Gibbs' free solvation energies of the solute transfer from *n*-octanol to water. A good overall agreement between DFT calculated and experimentally determined log*K*_OW_ values was established (*R* ^2^ = 0.8024–0.9658).

## 1. Introduction

Apart from being important in material science and catalysis, metal ions and their complexes play a significant role in the vital functions of living organisms. Numerous applications of metal-based compounds as both therapeutic and diagnostic agents as well as mineral supplements were studied in scope of relatively young but rapidly developing research discipline named medicinal inorganic chemistry [1]. Metal complexes have a long history of use as medicines with cytostatic, antirheumatic, or anti-inflammatory properties. Further, they have been used in treatment of cardiac and many other diseases, while the anticancer activity of metal complexes, especially of cisplatin related drugs, is of the greatest importance [2].

In addition to the widely used platinum-based chemotherapeutic drugs such as cisplatin or carboplatin, numerous non-platinum-based compounds were investigated as anticancer agents [1, 2]. Among them various ruthenium complexes attracted recent attention. Their anticancer activity, as well as clinical toxicity, is clearly distinct from platinum complexes [3–5]. Diversity of modes of action that involves both extra- and intracellular processes was achieved through interactions of ruthenium complexes with plasma proteins, extracellular matrix collagens, actins on the cell surface, regulatory enzymes in the plasma membranes or cytoplasm, and DNA in the cell nucleus [6]. In addition, some ruthenium complexes exhibit greater efficacy against cancer metastasis than against primary tumors by modulating adhesion, migration, invasion, proteolytic degradation of extracellular matrix, and new blood vessel formation. Over the last thirty years the interest for various ruthenium complexes, their synthesis and potential anticancer activity is constantly increasing, among them ruthenium(II)-arene compounds are being in the focus of research.

Our previous studies have been focused on syntheses, characterization, and cytotoxic activity of series of ruthenium(II)-arene compounds [7–9]. It has been shown that the presence of arene ligand is crucial. It is involved in several steps: it stabilizes the +2 ruthenium oxidation state, affects the cell uptake, influences the interactions with potential intracellular targets, and provides satisfactory lipophilicity needed to cross the cell membrane.

Since the lipophilicity is one of the major parameters affecting important biological processes that follow drug intake such as adsorption, passage through membranes, drug-receptor interactions, metabolism, and toxicity of molecules [10], we have decided to put an emphasis on the determination and estimation of lipophilicity of several ruthenium(II)-arene complexes in the scope of the present work.

Octanol-water partition coefficient (log⁡*K*_OW_) is a widely accepted measure of lipophilicity and can be determined for various compounds including metal complexes in several ways. According to original “shake-flask” method, *K*_OW_ is defined as a concentration ratio of compound distributed between* n*-octanol and aqueous phase. However, this time-consuming method with many experimental limitations has been replaced with chromatographic methods such as reversed-phase thin-layer chromatography (RP-TLC) [11], high-performance liquid chromatography (HPLC) in both, isocratic and gradient elution modes [12, 13], microemulsion electrokinetic chromatography (MEEKC) [14], and immobilized artificial membrane chromatography (IAM) [15]. Also, many software applications such as ALOGP, KOWIN, and CLOGP that incorporate different calculation approaches and algorithms have been frequently applied. However, these commonly used programs for log⁡*K*_OW_ estimation are not suitable in the case of complex compounds simply because the appropriate input of the central metal atom is usually missing, therefore resulting in poor predictions [16].

While considerable effort has been devoted to studying the log⁡*K*_OW_ of organic compounds, to the best of our knowledge, only few papers are dealing with the measure of log⁡*K*_OW_ of various complexes mostly by the means of the shake-flask method, HPLC in various reversed-phase modalities, and MEKC [16], probably because of the fact that complex compounds exhibit a very intricate behavior in liquid-liquid partitioning systems including HPLC that requires a delicate approach [17].

However, despite its numerous advantages and long history of successful application for lipophilicity determination of small organic molecules, modern high-performance thin-layer chromatography is rarely used for log⁡*K*_OW_ determination of complex compounds, except for a few cases [18].

On the other hand, modern computational approach to the estimation of lipophilicity of metal complexes mostly relies on quantitative structure property relationship studies (QSPR) that establish quantitative models based on experimentally determined log⁡*K*_OW_ data and *ab initio* calculated molecular descriptors, such as the case with numerous platinum-based complexes [19, 20]. However, complete, reliable, and comparable data which correlate the log⁡*K*_OW_ with relevant properties and molecular descriptors are needed. Molecular descriptors often reflect complex and multiple physical interactions, and model should include as many descriptors as possible, often leading to difficult and ambiguous interpretation of the results. Another approach based on *ab-initio* calculations is to estimate the free solvation energy change for the solute transfer from *n*-octanol to pure aqueous phase. Modern theoretical methods in quantum chemistry, such as density functional theory (DFT), possess great predictive power and in conjunction with continuum solvation models are proven to be very reliable in the determination of the free energy of solvation [21]. It is noteworthy to mention that advantages of DFT are particularly important for transition metal compounds [22, 23], although they are not always considered as innocent systems. Continuum solvation models are efficient tools for studying solvent effects on molecular structure, spectra, and energetics [24–27] and have been used with success for determination of partition coefficients [28], p*Ka* values [29], redox potentials [30], and so forth. Improvement of solvent models is active area in research in computational chemistry, and models are continually being improved and new versions and models frequently appear [31]. From a great variety of solvent models, in this work the universal model proposed by Marenich et al, based on Density (SMD) [32] is used due to the proven accuracy for first-principle calculation of solvation energies [33].

In the present work the attention was focused on the application of RP-TLC as a simple, fast, and reliable tool to determine log⁡*K*_OW_ values of ten ruthenium(II)-arene complexes with potential anticancer and antiproliferative activity. Moreover, there is a serious lack of information in the present literature about lipophilicity assessment of complex compounds by the means of TLC. In order to assure accuracy of the proposed methodology we have employed *ab-initio* DFT computational approach to calculate log⁡*K*_OW_ values from the differences in Gibbs' free solvation energies of the solute transfer from *n*-octanol to water and to compare estimated values with experimentally determined ones.

Being simple, fast and reliable RP-TLC provides retention data in the form of *R*_*F*_ and corresponding *R*_*M*_ values ((1) and (2)) that can be further used to derive several chromatographic descriptors for lipophilicity estimation: *R*_*M*_^0^, *b*, *C*_0_, and PC1. Consider the following:

(1) $$\begin{array}{c} R_{F}=\frac{x}{f}. \end{array}$$

Parameters *x* and *f* represent migration distance of the solute and solvent front, respectively. Consider the following:

(2) $$\begin{array}{c} R_{M}=\log\left(\frac{1}{R_{F}}-1\right), \end{array}$$

(3) $$\begin{array}{c} R_{M}=R_{M}^{0}+b\varphi . \end{array}$$

The first parameter (*R*_*M*_^0^) represents the intercept in the linear relation between retention of a solute, *R*_*M*_, and volume fraction of organic-mobile-phase modifier, *φ* (3). As a value extrapolated to 0% v/v of organic solvent it accounts for partitioning of a solute between pure water and the nonpolar stationary phase [34].

Slope, *b*, indicates the rate at which the solubility of the solute increases with changes in the mobile-phase composition and it is related to the specific hydrophobic surface area of a solute molecule [35]. Parameter *C*_0_, introduced by Bieganowska et al. [36], represents the ratio of *R*_*M*_^0^ and *b* and is considered as a concentration of organic modifier in the mobile phase for which the distribution of the solute between the two phases is equal.

The last descriptor, the first principal component, PC1, is derived from principal component analysis (PCA), that is, principal component regression (PCR), multivariate chemometric methods often applied on chromatographic data. It has been demonstrated that the scores of the principal components (usually the PC1 scores are sufficient) are better correlated with log⁡*K*_OW_ since PCA combines all chromatographic data in one single feature [37].

## 2. Experimental

### 2.1. Materials

**Figure 1:**
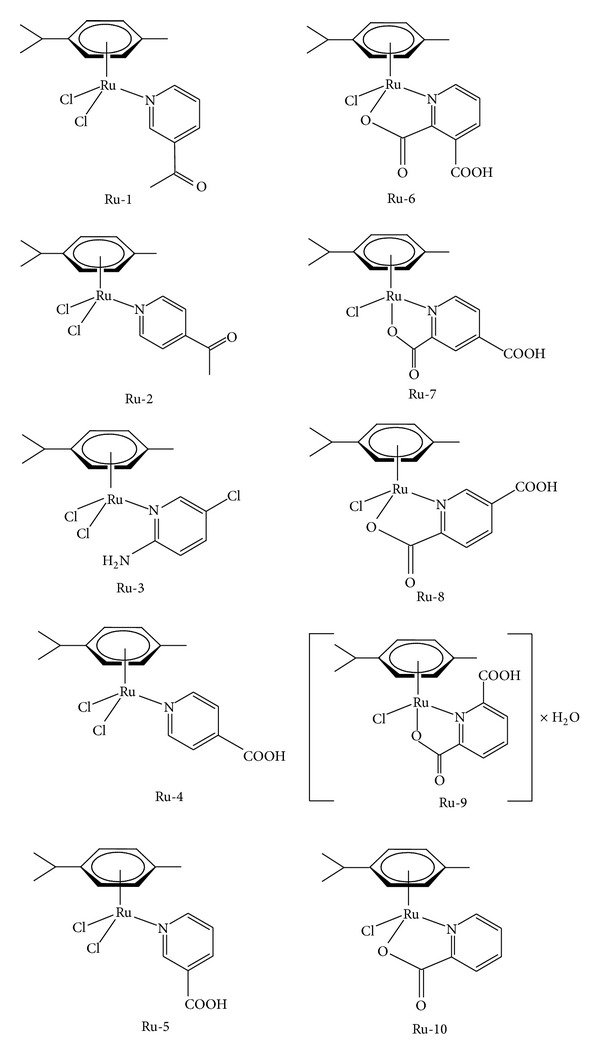
Structures of studied ruthenium(II)-arene complexes.

All reagents and solvents, including standard substances, were of analytical or HPLC purity grade. They are purchased from commercial suppliers: Aldrich (Milwaukee, WI, USA), Fluka (Buchs, Switzerland), and Merck (Darmstadt, Germany), and used as received. Ruthenium(II)-arene complex compounds (Figure 1) were synthesized and characterized as already described [7, 8].

In the present work a set of 28 standard solutes, mainly mono- and polysubstituted phenols, aromatic carboxylic acids, ketones, amines, esters, and few polyaromatic hydrocarbons, of known log⁡*K*_OW_ values (Table 1) were chosen for calibration. Experimentally determined values of log⁡*K*_OW_ were taken from the KOWWIN software (EPI Suite, v. 4.11 US EPA). The set of standard compounds was compiled considering that hydrogen bonds, for both proton accepting and proton donating, as well as dipolar interactions, among the overall interactions that solutes exhibit in a chromatographic environment, should be present in a way as to ensure the ability of the final model to describe the behavior of a diverse set of compounds. The optimal range of log⁡*K*_OW_ values was considered broad enough to provide a reliable regression performance (from 1 to 5 log⁡*K*_OW_ units). Dissociation constants for ionogenic compounds were collected from several sources and presented along with the calculated degree of ionization (*α*) at pH = 6 in Table 1.

### 2.2. Chromatographic Procedure

For all chromatographic experiments solutions of standard substances, as well as of studied ruthenium complex compounds, were prepared by dissolving appropriate amount of substance in acetone in concentration of 0.1 mg/mL. Commercially available octadecyl modified silica aluminum sheets (Art. number 5559, Merck, Darmstadt, Germany) were cut into 10 × 10 cm plates. The plates were manually spotted with approximate volume of 1.0 *μ*L of freshly prepared solutions, at 5 mm distance from each edge of the plate. Mixtures of acetonitrile and water were used as mobile phases, with organic component content increasing in the range of 30–60% v/v, with increment of 5%. Other organic modifiers such as methanol and acetone have been tested, but some of the ruthenium complexes were either moved through the system nonselectively or completely retained at the starting points. They are, therefore, discarded from the further study. The chromatograms were developed using horizontal developing chamber (CAMAG, Mutenz, Switzerland). The solvent migration distance was about 4.5 cm. The plates were visually inspected under the UV light (254 nm) and each zone was clearly marked and its distance was manually measured. All measurements were done in triplicate and average values were used in further calculations. All experiments were performed at ambient temperature (22 ± 2°C).

### 2.3. Calculation of Chromatographic Descriptors

The following chromatographic descriptors: *R*_*M*_^0^, *b*, and *C*_0_, were calculated based on the retention data (Table 2). *R*_*M*_^0^ and *b* were obtained as the intercept and slope, respectively, according to (3). The hydrophobicity parameter *C*_0_ was calculated as the ratio (−*R*_*M*_^0^/*b*). However, this parameter did not show promising retention—log⁡*K*_OW_ models, and was excluded from the further study. All necessary calculations were performed using the Data analysis tool-pack (Microsoft Excel 2010). The obtained models are summarized in Table 3 followed by accompanying statistics.

### 2.4. Modeling and Validation of TLC Retention—log⁡*K*_OW_ Relationship

Calibration models based on individual *R*_*M*_ values as well as extrapolated *R*_*M*_^0^ and *b* descriptors were established using standard set of compounds (Table 1) and ordinary least squares procedure (Data analysis tool-pack, Microsoft Excel 2010). Principal component regression was employed instead of separate calculation of PC1 score values, using singular value decomposition (SVD) algorithm, followed by random subset cross-validation (five splits and one iteration) procedure. All calculations were made with PLS Tool Box v. 7.2., (Eigenvectors Research Inc.) for MATLAB R2011a (Mathworks Inc.). Quality of obtained models was assessed by the means of Pearson's *R* ^2^ and accompanying statistics.

### 2.5. DFT Computational Details

The common logarithm of *K*_OW_ is calculated as

(4) $$\begin{array}{c} \log K_{\text{OW}}=\frac{\Delta G_{\text{sol}(\text{water})}-\Delta G_{\text{sol}(\text{oct})}}{2.303RT}, \end{array}$$

where Δ*G*_sol_ is the standard state solvation free energy of a given complex in octanol (oct) or in water (water) at *T* = 298 K. The standard-state solvation free energy is defined as the free energy of transfer from the gas phase to the condensed phase under standard state conditions. Because the gas-phase free energies are calculated with respect to a standard state of 1 atm, a correction factor of *RT*ln⁡(24.46) (1.894 kcal mol^−1^ at 298 K) needs to be added to convert it into the standard state of 1 mol dm^−3^.

All the DFT calculations have been carried out with the Gaussian 09, revision C.01 electronic structure program suite [38]. The local density approximation characterized by the Vosko-Wilk-Nusair (SVWN5) parametrization [39] has been used for the gas phase geometry optimizations, proven to be accurate for geometries of Werner type complexes [40–42]. The ruthenium cation was described by LANL2DZ relativistic effective core potentials (ECP) that replace 28 core electrons with a nonlocal effective potential and associated basis set for remaining electrons [43]. 6-31G+(d,p) basis set [44] was used for all other atoms. For the determination of log⁡*K*_OW_, M06L density functional [45, 46], with the ECP LANL2TZ basis set [47] for ruthenium, and 6-311+G(2d,p) for other atoms were used. The free energies of solvation were calculated using continuum solvation model based on density (SMD) [32]. With SMD, the 298 K solvation Gibbs energy is defined as the difference between the solvent and gas electronic energies [48], necessitating corresponding gas-phase calculation.

## 3. Results and Discussion

For all studied compounds a good linearity between retention constants (*R*_*M*_) and the volume fraction of acetonitrile was obtained resulting in well-established chromatographic descriptors (*R*_*M*_^0^ and *b*) (Table 3), even in the case of strongly polar or ionizable compounds as well as highly hydrophobic solutes. Although *C*_0_ was initially calculated, this parameter was later excluded from further consideration, because of statistically significant, but poor, correlation with log⁡*K*_OW_ values (*R* ^2^ = 0.4978, *P* < 10^−4^).

All calibration models were obtained using entire set of standard compounds. Accompanying statistics and equations are summarized in Table 4. Depending on the particular model, some of standard compounds were identified as outliers, with borderline statistical significance. However, all data were kept because of lack of reasonable justification for outlier removal. The best model was achieved using *R*_*M*_^0^ parameter (*R* ^2^ = 0.8857), while direct calibration of retention in 55% acetonitrile-water system (model no. 4) showed the worst correlation (*R* ^2^ = 0.6521). There is obvious deterioration in statistical performance of calibration models based on *R*_*M*_ values with an increase of acetonitrile content in mobile phase; however, with exception of the model number 4, no model can be statistically justified as being preferred over the others. Use of direct calibration is more convenient compared to calculation of *R*_*M*_^0^, *b*, and PCR since it does not require additional experimental and computational work. However, *R*_*M*_^0^ and *b* can be assessed for both highly lipophilic and polar compounds that exhibit measurable retention (0.2 < *R*_*F*_ < 0.8) in different range of organic modifier volume ratios, in contrast to direct calibration based on isocratic chromatographic conditions. Because of its physical meaning, *R*_*M*_^0^ parameter might be the most suitable.

The calculated gas-phase geometries of all complexes under investigation are in excellent agreement with available X-ray crystal structures [7, 8] (Table 5).

Chromatographically determined log⁡*K*_OW_ values were obtained using retention data (Table 2) and established calibration models (Table 3). Data are summarized in Table 6. All estimation methods (models 1–7) give mutually coherent data, with no statistical difference in between. Also there is a good overall agreement between DFT calculated and experimentally determined values (*R* ^2^ = 0.8024–0.9658).

Studied ruthenium(II)-arene complexes exhibit unusually high lipophilicity, in the range of 1–4 log units, compared with reported log⁡*K*_OW_ values of different series of platinum(II) and platinum(IV) complexes [19, 20] as well as with some Ru-(*η* ^6^-arene) compounds of similar structure that show significantly low octanol-water distribution coefficients [49]. However, all mentioned compounds have been positively charged, which might explain their low lipophilicity in comparison with neutral complexes investigated in current study.

## 4. Conclusion

The lipophilicity of ten ruthenium(II)-arene complexes with potential anticancer and antiproliferative activity has been determined by means of RPTLC on RP-18 silica as stationary and binary acetonitrile-water solvent systems as mobile phase. Based on retention data corresponding chromatographic descriptors for lipophilicity assessment have been obtained. Most of the experimental findings described above have been confirmed by DFT free energy calculations of complexes in octanol and water as solvents, using solvation model based on density (SMD). As results are promising, it can be considered as reliable tool for prediction of lipophilicity and rational design of coordination compounds with desired properties.

## Figures and Tables

**Table 1:** Calibration set of standard compounds with experimentally determined log⁡*K*_OW_ values.

No.	Compound	log⁡*K*_OW_	p*Ka*	*α* Ionization degree (%)
1	1,2,3-Benzotriazole	1.44	8.37	0.42
2	4-Chlorobenzoic acid	2.65	3.98	99.05
3	2-Nitrobenzaldehyde	1.74	—	—
4	4-Bromoaniline*	2.26	3.86	99.28*
5	Phenol	1.46	10.09	0.01
6	Benzophenone	3.18	—	—
7	3-Nitrobenzaldehyde	1.46	—	—
8	4-Aminobenzoic acid	0.83	4.65	95.72
9	Phthalimide*	1.15		100.00*
10	1,4-Benzoquinone	0.20	—	—
11	4-Nitrophenol	1.91	7.15	6.61
12	3-Nitrophenol	2.00	8.28	0.52
13	Benzyl alcohol	1.10	—	—
14	Naphthylamine*	2.25	3.92	99.18*
15	2-Naphthol	2.85	9.67	0.02
16	4-Fluoroaniline*	1.15	4.70	95.23*
17	1-Naphthol	2.85	9.34	0.05
18	4-Hydroxybenzaldehyde	1.35	—	—
19	3-Chloronitrobenzene	2.46	—	—
20	2,4-Dichlorophenol	3.06	7.85	1.39
21	4-Methylphenol	1.94	10.09	0.01
22	4-Chlorophenol	2.39	9.38	0.04
23	Anthracene	4.45	—	—
24	Acetophenone	1.58	—	—
25	2-Aminophenol	0.62	9.44	0.04
26	4-t-Butylphenol	3.31	10.31	0.00
27	1,3,5-Trihydroxybenzene	0.16	9.40	0.04
28	2,6-Dimethylphenol	2.36	10.59	0.00

*Calculated to the corresponding protonated conjugated acid.

**Table 2:** Retention of standard compounds and studied ruthenium complexes given as *R*_*F*_ values.

Number	Compound	Volume fraction of acetonitrile (%)						
		30	35	40	45	50	55	60
1	1,2,3-Benzotriazole	—	—	0.31	0.39	0.48	0.51	0.53
2	4-Chlorobenzoic acid	—	—	0.13	0.16	0.22	0.24	0.38
3	2-Nitrobenzaldehyde	—	—	0.11	0.14	0.21	0.24	0.30
4	4-Bromoaniline	—	—	0.06	0.08	0.15	0.18	0.25
5	Phenol	—	—	0.26	0.33	0.40	0.50	0.57
6	Benzophenone	—	—	0.03	0.05	0.07	0.09	0.15
7	3-Nitrobenzaldehyde	—	—	0.11	0.14	0.23	0.24	0.30
8	4-Aminobenzoic acid	—	—	0.44	0.48	0.54	0.56	0.60
9	Phthalimide	—	—	0.24	0.30	0.41	0.43	0.48
10	1,4-Benzoquinone	—	—	0.40	0.43	0.49	0.50	0.52
11	4-Nitrophenol	—	—	0.24	0.28	0.33	0.46	0.52
12	3-Nitrophenol	—	—	0.16	0.20	0.27	0.41	0.49
13	Benzyl alcohol	—	—	0.42	0.50	0.53	0.59	0.64
14	Naphthylamine	—	—	0.12	0.16	0.22	0.33	0.41
15	2-Naphthol	—	—	0.13	0.18	0.27	0.37	0.43
16	4-Fluoroaniline	—	—	0.26	0.28	0.36	0.48	0.52
17	1-Naphthol	—	—	0.12	0.13	0.19	0.33	0.39
18	4-Hydroxybenzaldehyde	—	—	0.41	0.39	0.48	0.61	0.63
19	3-Chloronitrobenzene	—	—	0.07	0.09	0.11	0.22	0.28
20	2,4-Dichlorophenol	—	—	0.09	0.12	0.27	0.33	0.40
21	4-Methylphenol	—	—	0.26	0.30	0.36	0.48	0.54
22	4-Chlorophenol	—	—	0.17	0.24	0.30	0.39	0.51
23	Anthracene	—	—	0.01	0.02	0.02	0.09	0.15
24	Acetophenone	—	—	0.23	0.26	0.31	0.41	0.51
25	2-Aminophenol	—	—	0.42	0.42	0.51	0.62	0.66
26	4-t-Butylphenol	—	—	0.07	0.09	0.16	0.28	0.39
27	1,3,5-Trihydroxybenzene	—	—	0.73	0.67	0.77	0.80	0.84
28	2,6-Dimethylphenol	—	—	0.11	0.16	0.23	0.33	0.41
29	Ru-1	0.01	0.01	0.02	0.04	0.06	0.11	—
30	Ru-2	0.00	0.01	0.02	0.04	0.06	0.12	—
31	Ru-3	0.01	0.01	0.02	0.04	0.06	0.15	—
32	Ru-4	0.01	0.02	0.05	0.07	0.10	0.14	—
33	Ru-5	0.01	0.03	0.06	0.08	0.13	0.16	—
34	Ru-6	0.08	0.13	0.19	0.24	0.31	0.37	—
35	Ru-7	0.23	0.28	0.32	0.37	0.44	0.49	—
36	Ru-8	0.11	0.15	0.23	0.27	0.33	0.45	—
37	Ru-9	0.05	0.07	0.13	0.16	0.21	0.29	—
38	Ru-10	0.02	0.04	0.07	0.11	0.19	0.24	—

**Table 3:** Linear relationship between solute retention (*R*_*M*_) and organic modifier volume fraction (*φ*). Accompanied statistics (*R*: Pearson's correlation coefficient, *F*: Fisher's parameter, S.D.: model standard deviation, *n*: number of calibration points) with values of intercept (*R*_*M*_^0^) and slope (−*b*).

Compound	Chromatographic parameters					
	*R*_*M*_^0^	−*b*	*R *	S.D.	*F *	*n *
1,2,3-Benzotriazole	1.13 ± 0.16	2.05 ± 0.32	0.9642	0.052	39.65	5
4-Chlorobenzoic acid	1.98 ± 0.18	2.83 ± 0.36	0.9770	0.056	62.92	5
2-Nitrobenzaldehyde	1.99 ± 0.11	2.73 ± 0.22	0.9901	0.035	149.91	5
4-Bromoaniline	2.74 ± 0.19	3.81 ± 0.38	0.9858	0.059	103.48	5
Phenol	1.64 ± 0.05	2.94 ± 0.11	0.9981	0.017	768.73	5
Benzophenone	2.86 ± 0.11	3.42 ± 0.23	0.9935	0.036	229.29	5
3-Nitrobenzaldehyde	1.98 ± 0.20	2.73 ± 0.39	0.9707	0.062	48.96	5
4-Aminobenzoic acid	0.65 ± 0.07	1.38 ± 0.14	0.9848	0.022	96.39	5
Phthalimide	1.40 ± 0.18	2.32 ± 0.35	0.9671	0.056	43.29	5
1,4-Benzoquinone	0.61 ± 0.08	1.10 ± 0.15	0.9723	0.024	51.88	5
4-Nitrophenol	1.66 ± 0.13	2.83 ± 0.25	0.9886	0.039	129.19	5
3-Nitrophenol	2.22 ± 0.15	3.67 ± 0.30	0.9900	0.048	147.99	5
Benzyl alcohol	0.87 ± 0.07	1.86 ± 0.13	0.9924	0.021	195.84	5
Naphthylamine	2.40 ± 0.08	3.75 ± 0.15	0.9976	0.024	612.44	5
2-Naphthol	2.30 ± 0.10	3.70 ± 0.21	0.9954	0.033	323.64	5
4-Fluoroaniline	1.56 ± 0.16	2.68 ± 0.32	0.9789	0.051	68.73	5
1-Naphthol	2.44 ± 0.23	3.75 ± 0.46	0.9785	0.072	67.56	5
4-Hydroxybenzaldehyde	1.16 ± 0.25	2.33 ± 0.49	0.9396	0.078	22.59	5
3-Chloronitrobenzene	2.67 ± 0.25	3.75 ± 0.50	0.9747	0.078	57.07	5
2,4-Dichlorophenol	2.73 ± 0.30	4.35 ± 0.60	0.9728	0.095	52.89	5
4-Methylphenol	1.61 ± 0.10	2.80 ± 0.194	0.9928	0.031	207.53	5
4-Chlorophenol	2.10 ± 0.08	3.49 ± 0.16	0.9969	0.025	487.62	5
Anthracene	4.37 ± 0.50	5.96 ± 0.98	0.9616	0.155	36.87	5
Acetophenone	1.66 ± 0.15	2.75 ± 0.29	0.9836	0.046	89.03	5
2-Aminophenol	1.15 ± 0.19	2.37 ± 0.37	0.9657	0.059	41.45	5
4-t-Butylphenol	3.16 ± 0.17	4.93 ± 0.35	0.9927	0.055	204.43	5
1,3,5-Trihydroxybenzene	0.38 ± 0.29	1.79 ± 0.57	0.8754	0.090	9.84	5
2,6-Dimethylphenol	2.41 ± 0.06	3.77 ± 0.13	0.9984	0.020	908.70	5
Ru-1	3.90 ± 0.32	5.45 ± 0.74	0.7903	0.155	6.65	6
Ru-2	3.97 ± 0.22	5.71 ± 0.51	0.8797	0.106	13.69	6
Ru-3	3.96 ± 0.16	5.75 ± 0.38	0.8263	0.079	8.61	6
Ru-4	3.24 ± 0.32	4.56 ± 0.74	0.9351	0.154	27.86	6
Ru-5	3.20 ± 0.30	4.69 ± 0.69	0.8732	0.145	12.84	6
Ru-6	1.97 ± 0.08	3.24 ± 0.18	0.9564	0.037	42.93	6
Ru-7	1.13 ± 0.08	2.02 ± 0.19	0.9875	0.039	157.10	6
Ru-8	1.88 ± 0.14	3.22 ± 0.31	0.9563	0.066	42.81	6
Ru-9	2.35 ± 0.17	3.58 ± 0.40	0.9010	0.084	17.25	6
Ru-10	3.04 ± 0.14	4.73 ± 0.31	0.9824	0.066	110.36	6

**Table 4:** Calibration models and accompanying statistics.

Model number	Model type	Equation	Statistics
1	OLS	*R*_*M*40%_ = (−0.17 ± 0.09) + (0.44 ± 0.04)log⁡*K*_OW_	*R* ^2^ = 0.8067, S.D. = 0.22, *P* < 10^−4^, *n* = 28
2	OLS	*R*_*M*45%_ = (−0.17 ± 0.08)+(0.38 ± 0.04)log⁡*K*_OW_	*R* ^2^ = 0.7882, S.D. = 0.20, *P* < 10^−4^, *n* = 28
3	OLS	*R*_*M*50%_ = (−0.32 ± 0.09)+(0.37 ± 0.04)log⁡*K*_OW_	*R* ^2^ = 0.7666, S.D. = 0.20, *P* < 10^−4^, *n* = 28
4	OLS	*R*_*M*55%_ = (−0.34 ± 0.09)+(0.29 ± 0.04)log⁡*K*_OW_	*R* ^2^ = 0.6521, S.D. = 0.15, *P* < 10^−4^, *n* = 28
5	OLS	*R*_*M*_^0^ = (0.3 ± 0.2)+(0.83 ± 0.06)log⁡*K*_OW_	*R* ^2^ = 0.8857, S.D. = 0.33, *P* < 10^−4^, *n* = 28
6	OLS	*b* = (1.2 ± 0.2)+(0.98 ± 0.08)log⁡*K*_OW_	*R* ^2^ = 0.8388, S.D. = 0.31, *P* < 10^−4^, *n* = 28
7	PCR	log⁡*K*_OW_ = 0.58 *R*_*M*40%_ + 0.63 *R*_*M*45%_ + 0.62 *R*_*M*50%_ − 0.95 *R*_*M*55%_	RMSEC = 0.375; *R*_Cal_^2^ = 0.8502
			RMSECV= 0.418; *R*_CV_^2^ = 0.8146

**Table 5:** Selected average bond lengths (Å) and valence angles (°) for available crystallographic data (a) and comparison with DFT energy-minimized structures (b).

Comp.	Bond lengths (Å)				Valence angles (°)			
	M–C	M–Cl	M–N	M–O	O–M–N	O–M–Cl	N–M–Cl	Cl–M–Cl
Ru-1								
a	2.18	2.40	2.13	—	—	—	86.0	87.0
b	2.19	2.37	2.05	—	—	—	84.8	88.7
Ru-7								
a	2.18	2.41	2.10	2.10	77.9	86.7	85.3	—
b	2.19	2.37	2.03	2.04	78.8	88.6	81.9	—
Ru-9								
a	2.19	2.40	2.16	2.09	76.9	83.7	85.2	—
b	2.18	2.37	2.04	2.03	79.1	86.6	82.8	—
Ru-10								
a	2.19	2.42	2.10	2.09	78.0	85.2	85.2	—
b	2.18	2.37	2.04	2.03	79.1	86.6	82.8	—

**Table 6:** Chromatographically determined and computationally estimated log⁡*K*_OW_ values of studied ruthenium complexes.

Comp.	Chromatographically determined							Estimated		
	Model number							Based on *ab initio* (DFT) computations		
	1	2	3	4	5	6	7	Δ*G*_sol_ (oct) (kcal/mol)	Δ*G*_sol_ (water) (kcal/mol)	log⁡*K*_OW_
Ru-1	4.26 ± 0.37	4.03 ± 0.38	4.19 ± 0.42	4.31 ± 0.56	4.31 ± 0.28	4.38 ± 0.34	3.72	−13.85	−19.81	4.36
Ru-2	4.13 ± 0.37	3.91 ± 0.37	4.09 ± 0.41	4.13 ± 0.55	4.39 ± 0.28	4.63 ± 0.35	3.65	−13.27	−19.35	4.45
Ru-3	4.13 ± 0.37	3.91 ± 0.37	4.09 ± 0.41	3.74 ± 0.52	4.38 ± 0.28	4.68 ± 0.36	3.97	−9.99	−17.63	5.59
Ru-4	3.42 ± 0.33	3.43 ± 0.35	3.50 ± 0.38	3.86 ± 0.52	3.52 ± 0.25	3.47 ± 0.30	2.91	−14.48	−19.60	3.75
Ru-5	3.18 ± 0.32	3.24 ± 0.34	3.08 ± 0.36	3.63 ± 0.51	3.47 ± 0.24	3.59 ± 0.30	2.63	−14.99	−19.97	3.65
Ru-6	1.81 ± 0.30	1.72 ± 0.32	1.81 ± 0.34	1.99 ± 0.45	2.00 ± 0.22	2.11 ± 0.27	1.63	−20.77	−23.24	1.81
Ru-7	1.15 ± 0.31	1.07 ± 0.33	1.15 ± 0.35	1.24 ± 0.46	0.98 ± 0.23	0.87 ± 0.28	1.14	−20.51	−22.57	1.50
Ru-8	1.61 ± 0.30	1.59 ± 0.32	1.68 ± 0.34	1.49 ± 0.45	1.88 ± 0.22	2.09 ± 0.27	1.78	−19.35	−21.61	1.66
Ru-9	2.33 ± 0.30	2.36 ± 0.32	2.39 ± 0.34	2.55 ± 0.46	2.44 ± 0.22	2.46 ± 0.27	2.14	−16.88	−19.70	2.06
Ru-10	2.99 ± 0.32	2.80 ± 0.33	2.56 ± 0.34	2.87 ± 0.47	3.27 ± 0.24	3.63 ± 0.31	2.59	−15.64	−19.15	2.57
